# Emerging Designs and Applications for Biomembrane Biosensors

**DOI:** 10.1146/annurev-anchem-061622-042618

**Published:** 2024-07

**Authors:** Ekaterina Selivanovitch, Alexis Ostwalt, Zhongmou Chao, Susan Daniel

**Affiliations:** Robert Frederick Smith School of Chemical and Biomolecular Engineering, Cornell University, Ithaca, New York, USA

**Keywords:** biomembrane, biosensor, cell-free protein synthesis, liposomes, supported lipid bilayers

## Abstract

Nature has inspired the development of biomimetic membrane sensors in which the functionalities of biological molecules, such as proteins and lipids, are harnessed for sensing applications. This review provides an overview of the recent developments for biomembrane sensors compatible with either bulk or planar sensing applications, namely using lipid vesicles or supported lipid bilayers, respectively. We first describe the individual components required for these sensing platforms and the design principles that are considered when constructing them, and we segue into recent applications being implemented across multiple fields. Our goal for this review is to illustrate the versatility of nature’s biomembrane toolbox and simultaneously highlight how biosensor platforms can be enhanced by harnessing it.

## 1. INTRODUCTION TO BIOMEMBRANE-BASED SENSORS

**Figure 1:**
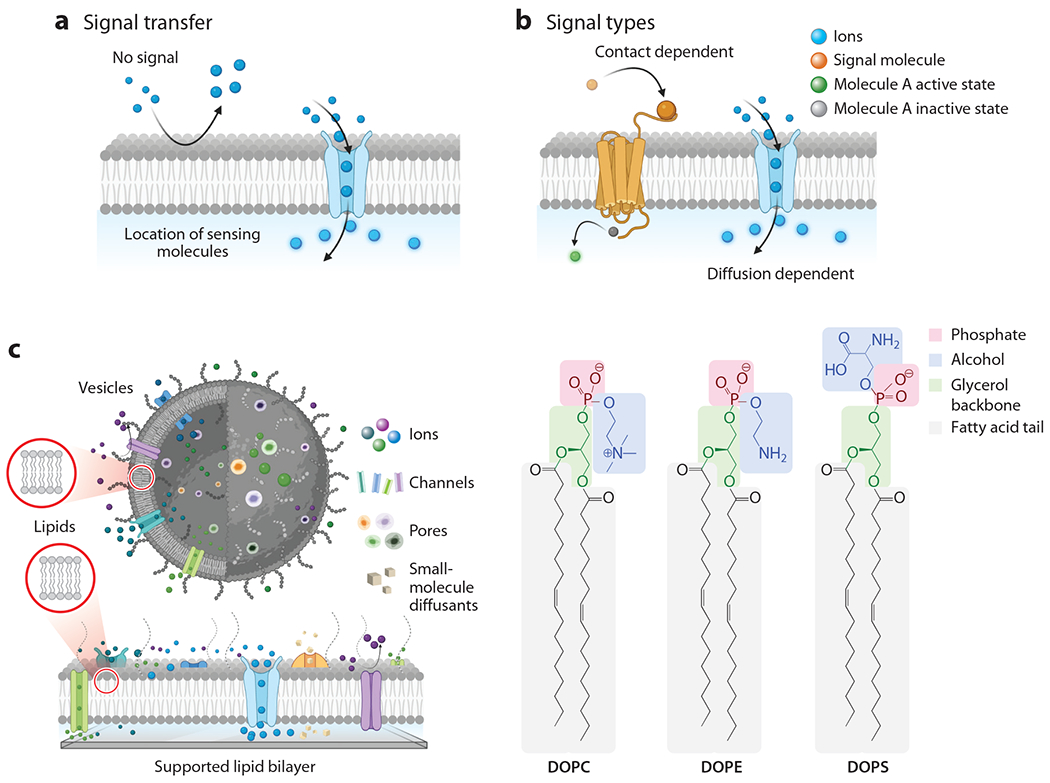
(*a*) Illustration of a membrane protein facilitating interactions between different environments via pore opening. (*b*) Illustration of a membrane protein facilitating either contact-based or diffusion-based signal transduction. (*c*) Structures of lipids composing vesicles or supported lipid bilayers into which various other biomacromolecules can be mixed. In a vesicle, events are transduced across the interior and exterior environments, while in a supported lipid bilayer, transduction spans across the bulk and supported membrane interfaces. Abbreviations: DOPC, dioleoylphosphatidylcholine; DOPE, 1,2-dioleoyl-sn-glycero-3-phosphoethanolamine; DOPS, dioleoylphosphatidylserine. Figure adapted from images created with BioRender.com.

Biological membranes are ubiquitous throughout nature and are critical features for survival and adaptation across all domains of life (i.e., prokaryotes, eukaryotes, archaea) and even some viruses. Although there is considerable variation between organisms, one of the main functions of these biomembranes, together with molecules embedded within, is to mediate communication between the environments on both sides of the membrane. For example, the cell plasma membrane (and its proteins) regulates transport of signaling molecules and ions across the membrane via passive and active processes and thus controls material that enters or exits the cell (1). Molecules can also pass across the membrane when proteoliposomes (e.g., synaptic vesicles, transport vesicles) bind to and fuse with the target membrane, releasing their cargo on the other side after the merger. However, the physical transport of material across the membrane is not the only communication pathway the cell uses (Figure 1a,b). Signals can also be transmitted across the membrane without physical transport of material when a molecule recognizes and binds to membrane receptors presented on one side. In this case, signal transduction across the membrane can occur by a few mechanisms when the transmembrane receptor responds to that binding event in some manner (through, e.g., a change in shape or chemical affinity). This response then induces downstream events on the other side of the membrane (Figure 1b) and thereby transduces the binding event occurring on one side of the membrane to the other. Even when the receptor itself does not undergo a physical or chemical change, a binding event to it can impact the local lipid organization and mobility surrounding the receptor and, in this manner, also signal a positive interaction at the binding surface. Being able to harness the ability of the cell to transduce signals across the membrane with such exquisite receptor binding specificity/selectivity—by fusion events, by controlled transport mechanisms, or by changes in lipid diffusion—has inspired the development of biomembrane-based biosensors, whose goals are to harness the cell’s communication capabilities for the design of biomimetic sensing platforms that eliminate the need for live cells (2–4).

Biomembrane sensors are now used as powerful detection platforms due to their component modularity, ability to self-assemble, biocompatibility, and adaptivity to various detection strategies. These features, along with many others, have made them invaluable in basic research pursuits and paradigmatic tools across a multitude of fields, including medicine (5–7), environmental science (8–10), and food safety (11–13). At a minimum, all biomembrane sensors require elements that facilitate specific recognition events, that generate measurable responses, and that detect signal output. Additional complexity and biosensing parameters can be afforded by modifying the chemical and physical properties, such as membrane composition and dimensionality, making them compatible with both two-dimensional (2D) surface-based and three-dimensional (3D) ensemble signal detection techniques. In this review, we cover recently developed membrane-based biosensors: their design, construction, and utility. We focus on those assembled from lipid vesicles and supported lipid bilayers (SLBs), which are suitable for ensemble and surface-based detection techniques, respectively, and summarize opportunities that are emerging in this rapidly developing biosensing field.

## 2. ESSENTIAL COMPONENTS FOR 2D AND 3D BIOMIMETIC MEMBRANES

In both SLB-based and vesicle-based sensors, the lipid bilayer is leveraged for its ability to support functional membrane biomacromolecules and to provide a physical barrier between the bulk environment and the sensor. These lipid bilayers can conveniently incorporate proteins to serve as receptors, signal transducers, or molecular transporters that receive specific stimuli to trigger measurable macromolecular responses. A notable feature of these membranes is the 2D mobility of the constituents, allowing receptors to rearrange to carry out native-like interactions, such as multivalent binding. Bilayers can also contain other synthetic or biologically derived constituents such as block copolymers and/or cholesterol or glycolipids that can support sensing function. Overall, the bilayer structure has a layered organization of charged/zwitterionic hydrophilic outer surfaces sandwiching a hydrophobic interior layer that accommodates transmembrane proteins that can, for instance, facilitate recognition or diffusion events. This unique 2D, proteolipid composite material facilitates interactions with bulk molecules surrounding it, other lipids and membrane components within it, and the sensing surface (Figure 1c) that translates these interactions into a measurable signal that humans can detect and interpret.

Phospholipids are the primary material component of these bilayers and consist of a hydrophilic phosphate head group, a hydrophobic hydrocarbon tail, and a glycerol backbone. Two of the glycerol’s alcohol groups are esterified with fatty acids, forming the tail, and the third is esterified with phosphoric acid, forming the head. The layered structure of a bilayer arises from the arrangement of these lipids in aqueous solutions into two opposing lipid tails and head groups facing out toward the bulk solution. Different chemical characteristics are afforded to the head group by further esterification of the phosphate group with an alcohol (Figure 1c). For example, the ester formed between a choline and the phosphate yields phosphatidylcholine: a neutral zwitterionic phospholipid at neutral pH, among the most common phospholipids found in mammalian cells, and the typical principal component of many biosensor systems. Other common phospholipids abundant in biological cell membranes, such as phosphatidylethanolamine and phosphatidylserine, are often included in membrane biosensors as well. The choice of phospholipids and the chemical features of their head groups contribute to the chemical and physical landscape of the resultant membrane surface, which can drastically affect molecular interactions and as such provide a powerful means to tailor the sensor for specific recognition events.

Interlipid interactions should also be weighted, as they can determine the arrangement of the bilayer upon self-assembly, its physical and chemical properties, its ability to incorporate proteins, and thus the eventual efficiency of the membrane sensor. For instance, dioleoylphosphatidylcholine and 1,2-dioleoyl-*sn*-glycero-3-phosphoethanolamine lipids, which differ in head group identity but share the same glycerol backbone, preferentially self-assemble into lamellar and non-lamellar phases, respectively, when mixed. For phase-separated bilayers, such as this example, the overall structure is lamellar, but composition-dependent changes in lateral pressure, dipole potential, and electric field profiles have been observed (14, 15). These features can be exploited for sensing purposes. In general, lipid composition is an important consideration in the assembly of biomembrane biosensors, as fluctuations in composition can affect, for instance, molecular permeation across the bilayer (16, 17), protein insertion and folding (15, 18), protein function (18, 19), and lipid–protein interactions (20, 21).

Many, although certainly not all, biomembrane biosensors incorporate membrane proteins to facilitate transactions between different environments. Selecting the appropriate protein for a sensor is highly dependent on the desired function. For example, receptor proteins can be incorporated into a lipid membrane to detect interactions between the receptor and another molecule, or they can facilitate molecular access to the sensor on the other side of the membrane. Ligand–receptor interactions should be very selective, if not specific, where a signal is generated upon formation of a specific ligand–receptor pair. A few examples of receptor-mediated sensor designs are presented below in this review.

When selecting a permeation-mediating protein, one should consider whether selectivity toward certain analytes is desired. For example, there are different pore proteins that facilitate size-dependent diffusion across a lipid bilayer, such as outer membrane proteins (OMPs) found natively in bacteria (e.g., OmpF and OmpC) (22). Biologically they have several important roles, including facilitating the transfer of nutrients into bacterial cells and mediating metabolic exchange (23). However, in the context of a biosensor, they can permit passive transport of hydrophilic molecules such as ions, antibiotics, and peptides, which can subsequently be sensed or used to promote a signal response (23–25). Another group of proteins consists of pore-forming proteins, which can be found in all domains of life, but some of the most prevalent examples are found in bacteria (e.g., hemolysin) and viruses (e.g., viroporins), with a few examples now being identified in eukaryotes (e.g., amyloid pores) (26). The biological functions of these are mostly considered toxic, or in other words, such proteins are released to induce cell death; however, with the tools of genetic manipulation and design, they can become incredibly useful tools in biomimetic membranes due to their adaptability to genetic engineering, robust assembly, ability to penetrate into membranes, and ability to transport small (*<*2-nm) molecules (27).

The examples above are for nonspecific transport across a membrane. There are also examples of pore-type proteins, termed channels or transporters, that allow for facilitated diffusion. In these instances, pores are typically in an opened or a closed conformation and are often referred to as gated (e.g., ligand gated, mechanically gated, voltage gated, light gated) and are typically more selective to the molecular diffusants. For instance, the TREK proteins are a family of mechano-, thermal-, and lipid-gated ion channels specific for the transport of potassium across a membrane (28). Voltage-dependent anion channels are another example of selective channels that favor anions (Cl^−^) in an open state and cations (K^+^, Na^+^) in a closed state (29). P2X2 is an example of a ligand-gated channel selective toward cations (30). These types of membrane proteins are especially useful for sensors that detect ions; a few examples are provided in the sections below.

Both soluble and membrane-associated proteins used in sensors are typically harvested and isolated from cell-based expression systems and then are reconstituted with lipid scaffolds, such as lipid vesicles and SLBs (31). The isolation of membrane proteins is more complicated than the isolation of proteins that are soluble, requiring procedures for precipitation and extraction from their native lipid environments and introduction into the new desired environment (i.e., sensor platform) (32). Such processes have been highly optimized and skillfully tailored to specific proteins such that their functions can be accessed more readily in vitro (33, 34). Nevertheless, there are still many membrane proteins for which these methods fail to preserve the required functionality, and therefore, we describe below in this review an emerging synthetic biology approach, termed cell-free protein synthesis (CFPS), as an alternate method to integrate membrane proteins into biomembrane sensors.

In the remainder of this review, we describe recently designed sensors using either lipid vesicles or SLBs as scaffolds. We first review how the membrane-based sensors are constructed and identify critical components required for generating a measurable signal response. In the case of lipid vesicle–based sensors, we focus mainly on component-based design strategies (e.g., sensor and other protein constituents), while for SLB-based sensors, we focus on technique-based design, as the type of detector heavily influences overall platform construction. We complete our review by describing the various fields that now employ these sensors for numerous applications.

## 3. DESIGN AND CONSTRUCTION OF LIPID VESICLE–BASED 3D SENSORS

Vesicle-based sensors have been designed to detect a variety of different analytes, such as ions and other small molecules. The minimal essential components of this kind of biomembrane sensor typically require the lipid vesicle, assembled from customizable lipid compositions, and the molecular sensor or reporter, often in the form of a protein or small molecule. This section focuses first on how these vesicles are assembled and tuned to incorporate the desired combinations of lipids and proteins, followed by the design parameters required for lipid vesicles to become sensors.

### 3.1. Common Vesicle Assembly Techniques

**Figure 2:**
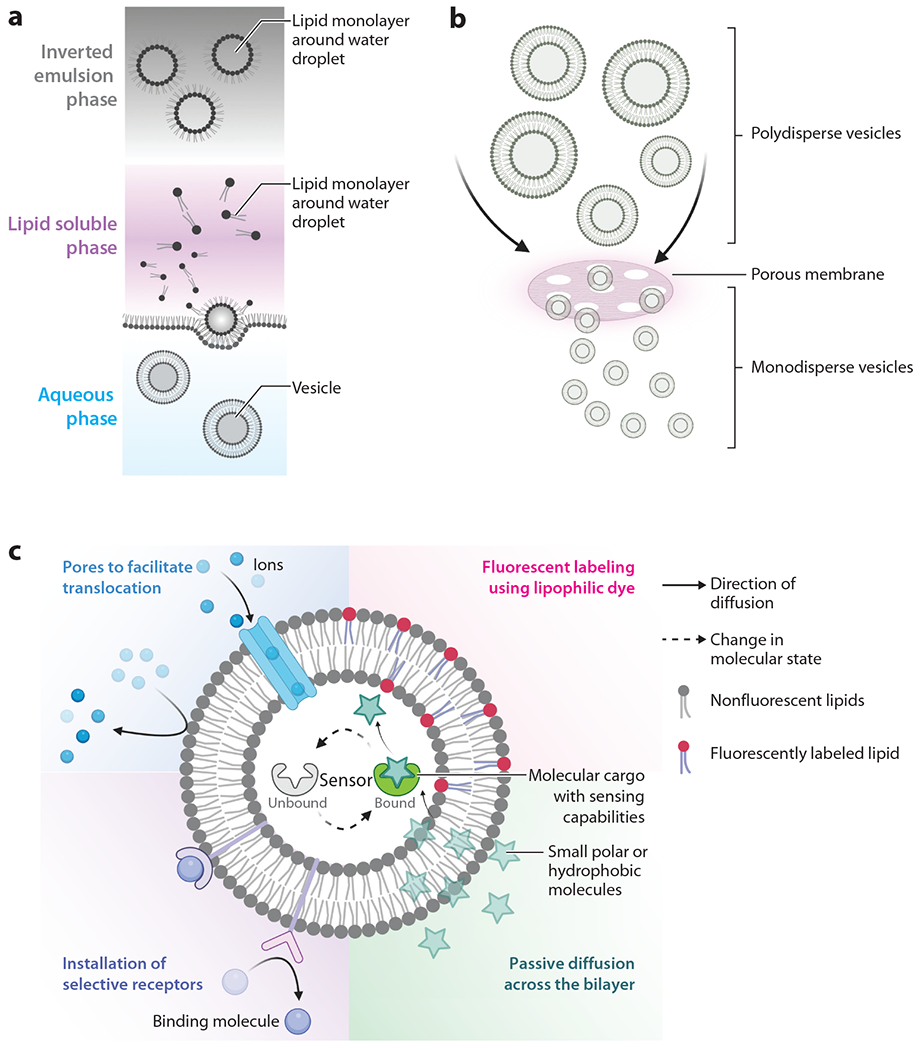
(*a*) Oil-in-water emulsion vesicle assembly method. (*b*) Extrusion vesicle assembly method. (*c*) Schematic drawing of different elements required for a vesicle-based biosensor in which diffusion can occur either passively across the membrane or via the assistance of membrane proteins. Sensors can be located within the interior of the vesicle or embedded within the bilayer to report interactions of analytes either at the membrane surface or upon accessing the interior environment. Figure adapted from images created with BioRender.com.

There are many techniques to assemble lipid vesicles and vesicle-based biosensors, which are described here in brief, but otherwise, we direct the reader to reviews and published works (35–38). One popular method is oil-in-water emulsion (39, 40), which uses phase separation to first assemble inverted emulsions (i.e., the head group faces the interior, and hydrophobic tails are exposed to the exterior); these will eventually form the inner vesicle leaflet (Figure 2a). Then a separate oil–water mixture is prepared in which the surfactants form a monolayer at the oil–water interface. As the inverted emulsion is added to the monolayer-containing solution, the droplets in the emulsion begin to sediment toward the interface, picking up a second layer and forming unilamellar lipid vesicles.

Another common method for vesicle preparation is the extrusion method (Figure 2b), in which lipids are first dehydrated, reconstituted in an aqueous solution, and subsequently extruded through a membrane filter of a defined size to form unilamellar and monodisperse vesicles (41). There are alternative methods when larger vesicles, such as giant unilamellar vesicles (GUVs), are desired. These larger GUVs can be produced by gentle hydration whereby an agarose gel is used as a solid support on top of which lipids are deposited, spread across the surface, and dried (42–44). The agarose–lipid hybrid is then slowly submerged in an aqueous buffer, and liposomes emerge due to forces normal to the lipid layer. As crowding increases at the surface, the liposomes begin to fuse with adjacent lipids, forming GUVs. A similar method was also developed using polyvinyl alcohol as the gel support (45). In octanol-assisted liposome (OAL) assembly, a relatively new technique that uses a microfluidic device, there are three stages: double-emulsion droplet formation, oil pocket formation within the bilayer, and solvent extraction (46). These assembly techniques offer gentle and biocompatible methods for constructing lipid vesicles that can incorporate user-defined membrane components beyond lipids, such as pore-forming proteins and sensors (Figure 2c).

### 3.2. Incorporating Additional Elements into Lipid Vesicles

Lipid vesicles offer two distinct environments for sensor and/or protein incorporation: the interior aqueous cavity, which can incorporate water-soluble components (e.g., proteins, soluble sensors), and the hydrophobic acyl chain region of the bilayer, which can incorporate water-insoluble molecules [e.g., transmembrane proteins, cholesterol (47), lipophilic dye] (48, 49). Cargo present in the aqueous phase can be encapsulated within the vesicle interior when it is assembled, regardless of the chosen assembly methods (described in Section 3.1). When hydrophobic molecules are being incorporated into the bilayer, they can be initially combined with lipids or, in some cases, added post-assembly, at which point they can partition into the bilayer. Dyes, fluorescent molecules, and even fluorescence resonance energy transfer (FRET) pairs are often integrated into the bilayer to allow for easy detection and tracking of the lipid vesicles (50). There are also examples of adding synthetic cross-linking reagents to the vesicle compositions, such as derivatives of polydiacetylene (PDA) (e.g., 10,12-tricosadiynoic acid), which offer unique color transitions in response to environmental cues (discussed below in Sections 3.3 and 5.1.2) (51–53). In the case of PDA, the membrane itself becomes the sensor, and no additional components are needed. Choosing the right membrane contents and interior cavity components is critical for the three basic element sensors discussed in the subsequent sections: facilitating recognition events using the bilayer constituents; generating a measurable response via a recognition event; and, finally, detecting the output signal.

### 3.3. Design of Vesicle Sensors

**Figure 3:**
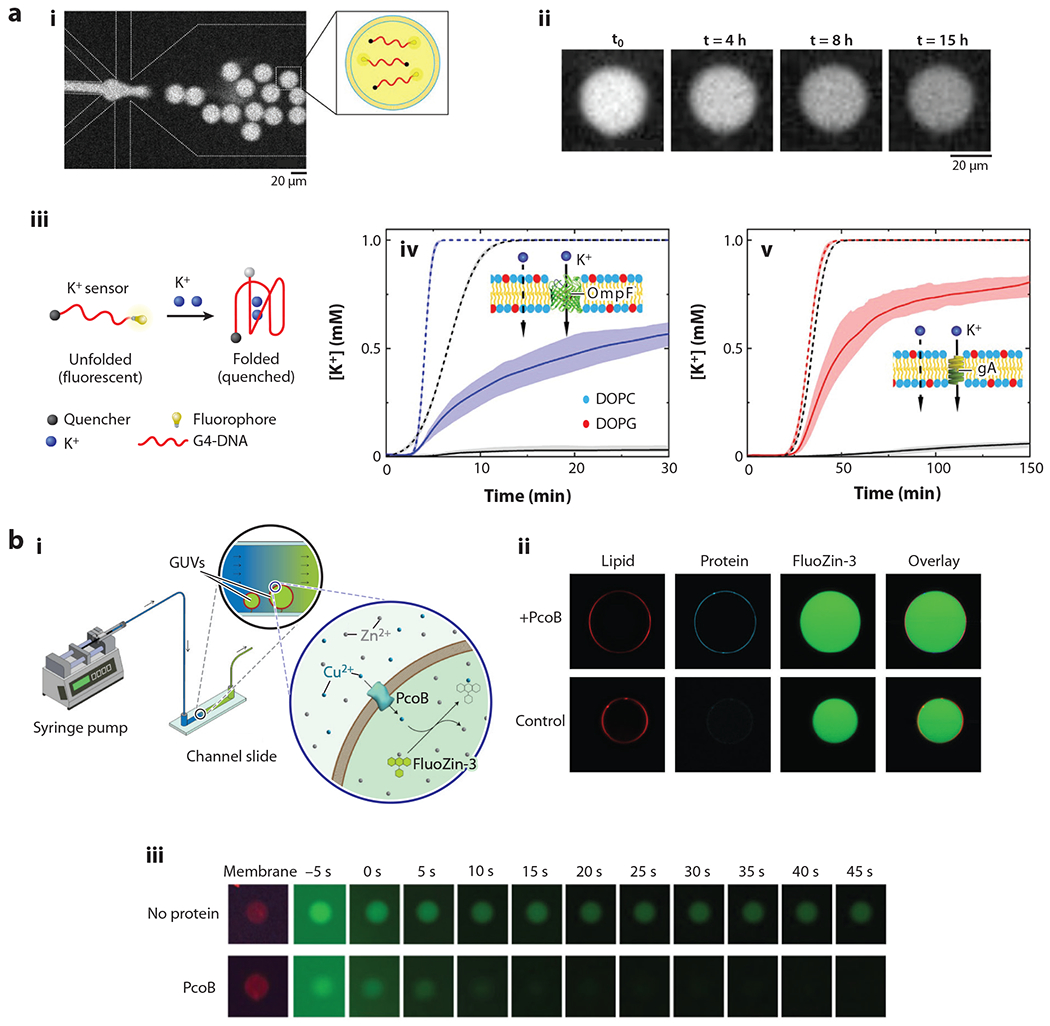
(*a*, *i*) Chip used for octanol-assisted liposome assembly. (*ii*) Time-lapse images of a sensing platform detecting potassium. As more potassium is sensed, there is a decrease in fluorescence. (*iii*) Representations of sensor function. (*iv*, *v*) Time-resolved studies with either (*iv*) OmpF-assisted (*blue solid line*) or (*v*) gramicidin-assisted (*red solid line*) diffusion compared to free diffusion, in which the sensor is not encapsulated (*dashed lines*). Black traces depict the absence of either OmpF or gramicidin. Panel *a* adapted from Reference 56 (CC BY 4.0). (*b*, *i*) Schematic drawing of a microfluidic chamber setup for detecting ion flux into GUVs. (*ii*) Images of an encapsulated copper ion sensor (FluoZin-3) in GUVs with and without the membrane protein porin. (*iii*) Time-lapse images of Cu^2+^ accessing the interior causing a fluorescence decrease versus when Cu^2+^ cannot access the interior and fluorescence remains the same. Panel *b* adapted from Reference 58 (CC BY 4.0). Abbreviations: DOPC, dioleoylphosphatidylcholine; DOPG, 1,2-dioleoyl-*sn*-glycero-3-phospho-(1-ac-glycerol) (sodium salt); FluoZin-3, fluorescent zinc indicator; GUV, giant unilamellar vesicle; PcoB, plasmid-encoded copper resistance outer membrane protein.

Most recent vesicle-based sensors have been designed for optical/spectroscopic readouts, such as fluorescence and absorbance. However, the general sensor design can be tailored to fit other readout systems such as those that are electrochemical (54, 55). One way to design a successful sensor into a vesicle is to encapsulate sensing molecules within the interior cavity and to limit molecular access to just the desired analyte. Upon a recognition event between the analyte and sensor, a measurable response is generated. For this to be successful, neither the sensor nor other bulk components can diffuse across the lipid bilayer, and neither element can be measurably active prior to the binding event (Figure 2c). This has been demonstrated by Fletcher et al. (56), who used the OAL method to encapsulate a potassium sensor inside GUVs and to install passive diffusion conduits (either gramicidin A or OmpF) into the lipid bilayer to facilitate transport into the vesicles (Figure 3a). This technique allowed these researchers to quantitively monitor flux into the vesicle and monitor fluorescent readouts across a range of wavelengths. A potassium sensor was also designed by the Kamat group, which integrated an ion-selective ionophore, valinomycin, into the bilayer and encapsulated a potassium-binding benzonfuran isophthalate probe that can detect potassium ions over sodium ions (57). Valinomycin facilitates passage across the bilayer by forming a macromolecular complex with potassium bound to its inner cavity, which is incompatible with sodium, thus limiting molecular access to potassium and other similar ions. A similar copper (Cu^2+^) sensor was designed using immobilized GUVs and a membrane-impermeable Cu^2+^ sensor, FluoZin-3 (58) (Figure 3b). These studies provide a general design overview for constructing a biomembrane sensor platform in which the detected analyte can randomly (such as through gramicidin A or OmpF) or selectively (such as described for valinomycin) diffuse across the membrane to access the sensing molecule inside.

In other instances, the sensing molecules can be embedded into the lipid bilayers where diffusion across the bilayer is not required, such as bilayers assembled with PDA lipids. PDA lipids contain conjugated (ene-yne) backbones and have a characteristic absorption peak at 640 nm upon polymerization. A colorimetric response (red to blue) can be detected as these lipids interact with specific molecules (59, 60). This approach has been especially advantageous when detecting and identifying analytes like antimicrobial peptides produced by bacteria (53), bacteriocins (61), and carbon dioxide (62).

Careful consideration needs to be taken when designing the lipid content for these vesicles to prevent nonspecific molecular mechanisms such as vesicle–vesicle or vesicle–cell fusion. In biology, these mechanisms are typically triggered and directed by fusion proteins that mediate docking and merging of two membranes (63); however, artificial vesicle fusion can proceed via electrostatically driven interactions (64, 65). Although fusion mechanisms were programmed into the design of both cited works, these interactions indicate that a given electrostatic landscape can result in unintentional access to the sensor and a false signal. Incorporating the sensing element into the bilayer is not limited exclusively to PDA lipids. Examples such as those incorporating FRET pairs (66, 67), quenched fluorophores (68), and split green fluorescent protein (GFP) (69) are among the many other examples in which the sensing molecules are embedded into the membrane, rather than encapsulated within.

These studies present a variety of different options for designing and integrating vesicle-based sensors; however, the scope of membrane-integrated proteins is limited to either stable membrane proteins, which can withstand standard isolation and are not disrupted in the absence of a membrane, or proteins that are amenable to reconstitution procedures. In an effort to circumvent these limitations, cell-free synthesis approaches are emerging for membrane proteins and are expanding the library of available membrane proteins that can be integrated into biomembrane sensor applications.

### 3.4. Emerging Techniques in Vesicle Sensor Design

CFPS combines the transcription (polymerase, transcription factors, etc.) and translation (ribosome, initiation factors, etc.) machinery together with the necessary building blocks (amino acids, salts) to synthesize a protein outside of a living cell. The emerging approach of using CFPS applied to transmembrane species has facilitated the in vitro expression of biomacromolecules into lipid bilayers, negating the laborious need to isolate them from cells first. There are plenty of available resources chronicling the development of CFPS (70–72); here we focus on the role of CFPS in the development of biosensors.

Riboswitches are a class of biomolecules that have drawn much attention in the biosensor realm. They are stretches of RNA that precede the translation start site and are used in bacteria to up- or downregulate gene expression, at either the transcriptional or translational level, in direct response to ligand binding (73). In other words, if a molecule (ligand) is bound, the aptamer domain of the RNA is tightly folded around it, and thus the downstream start site is not available for ribosomal binding. In the absence of the ligand, the RNA undergoes allosteric changes in structure that render the ribosomal binding site accessible. This property was exploited in synthetic systems in which reporters (i.e., GFP, yellow fluorescent protein) were installed downstream of the riboswitch to indicate successful transcription or translation when fluorescence appeared. In one instance, a transcriptionally regulated riboswitch was encapsulated within lipid vesicles to detect fluoride ions (74). By using the oil-in-water emulsion method, Boyd et al. (74) were able to form lipid vesicles enclosing the CFPS mixture and a DNA strand encoding for a riboswitch variant. When fluoride ions were present, they diffused across the semipermeable membrane, bound to the riboswitch’s aptamer, and allowed for the transcription of the downstream reporter (GFP). In the absence of fluorine, the riboswitch folded into a different conformation, exposed its transcription terminator sequence, and therefore inhibited transcription of the reporter. In a similar encapsulation design, a translationally regulated riboswitch to detect theophylline—a carefully monitored clinical drug—has been demonstrated (75). Accordingly, integrating riboswitches with vesicle sensor designs allows for an easily detectable optical signal upon the generation of the reporter signal, while the library of available aptamers can potentially expand the applicability of this design to many targets in the future.

Riboswitches are not the only sensing elements that can be incorporated into vesicles. Adamala et al. (76) showed that genetic circuits, whose underlying mechanisms are similar to those of riboswitches, can be successfully encapsulated using the extrusion method and then used to detect both membrane-permeable and membrane-impermeable small molecules. They used transcription inducers, such as Dox, IPTG, and arabinose, to regulate reporter gene expression and translocation protein (Tet) and the pore-forming protein alpha-hemolysin for shuttling molecules across the lipid membrane. By carefully tuning both the inducer and the pores, they showed that reported gene expression can be tightly regulated and triggered only in the presence of desired molecules.

There are many design parameters to consider for customizing vesicle-based sensors, such as the location of the sensory element (i.e., inside the vesicle or within the bilayer), the type of signal readout (i.e., direct measurable response to binding or detection of downstream event), and whether additional elements (i.e., channel or pore proteins) need to be included to improve sensor selectivity. Nevertheless, there are many potential vesicle sensor designs that have yet to be explored, and we anticipate that with the growing tools of synthetic biology at hand, novel approaches will continue to emerge.

## 4. CONSTRUCTION AND DESIGN OF SUPPORTED LIPID BILAYER SENSORS

**Figure 4:**
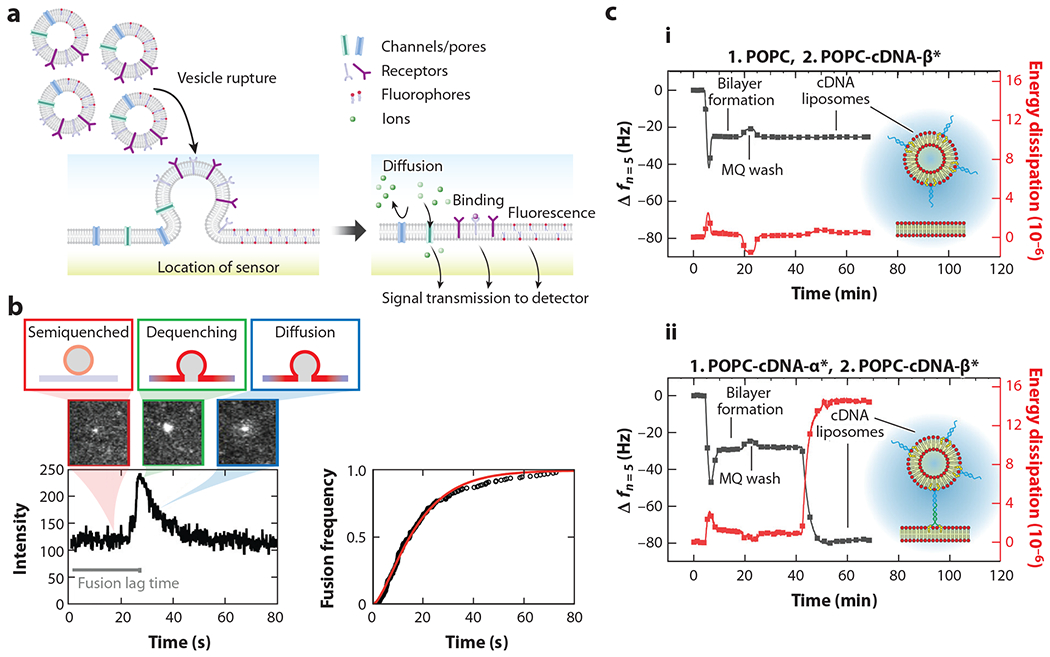
(*a*) Illustration of SLB formation from lipid vesicles via the vesicle fusion method and the general categories of biological events enabled by SLB-based biosensors. Vesicle fusion is perhaps one of the most widely used, accessible, and simple methods for forming SLBs. (*b*) A typical single virus fusion event captured by TIRF microscope. Panel *b* adapted with permission from Reference 80; copyright 2023 American Society for Microbiology. (*c*, *i*) QCM-D signal of SLB formation on a sensor without the tethering of microbubbles. (*ii*) QCM-D signal of SLB formation on a sensor followed by the tethering of microbubbles to SLBs. Panel *c* adapted from Reference 81 (CC BY 4.0). Abbreviations: POPC, 1-palmitoyl-2-oleoyl-glycero-3-phosphocholine; QCM-D, quartz crystal microbalance with dissipation monitoring; SLB, supported lipid bilayer; TIRF, total internal reflection.

SLBs are planar lipid membranes formed on solid supports and provide an alternative geometry to spherical lipid vesicles for studying membrane-mediated processes. Common methods to assemble SLB-based sensors from lipid vesicles include vesicle fusion (Figure 4a), solvent-assisted lipid bilayer assembly, and Langmuir–Blodgett/Langmuir–Schaefer steps. Many excellent reviews discuss SLB formation methods (77–79); therefore, we focus here on SLBs used specifically for biosensing applications.

To take advantage of the planar structure of SLBs, the bottom leaflet of an SLB can be designed to assemble on many different surface types, which expands the possibilities for sensing modalities. This flexibility means that the chemo-, thermo-, or mechano-responses of receptors, ion channels, pore-forming proteins, and other biomolecules in the membrane can be assessed by a variety of approaches. In general, there are three common sensor readout approaches for SLBs: acoustical, optical, and electrical.

### 4.1. Supported Lipid Bilayer Sensors: Acoustic Detection

Quartz crystal is a natural piezoelectric material in which mechanical deformation leads to changes in its electrical properties and vice versa, making it an excellent sensor capable of reporting changes occurring within an SLB. The experimental approach of using this piezoelectric property for sensing is referred to as a quartz crystal microbalance (QCM). QCM consists of two electrodes on opposite sides of a thin quartz crystal disk, where an alternating voltage can be applied to oscillate the quartz crystal disk at its natural resonance frequency and corresponding overtones. The adsorption of mass on the crystal, such as the rupturing of vesicles to form an SLB or the binding of an analyte to a receptor in the SLB, results in a shift in resonance frequency (Δ *f*) that correlates with the change of mass (Δ*m*) on the quartz crystal disk, as shown by the Sauerbrey equation:

$$\Delta m=-C*\frac{\Delta f}{n}.$$

In the equation above, *C* is the mass sensitivity constant related to the properties of quartz, while *n* is the overtone number. Kanazawa & Gordon (82) made viscosity corrections to the resonance frequency shifts to account for different liquid environments. In such cases, the shift is directly proportional to liquid density and viscosity while inversely proportional to quartz density and shear modulus. This correction to resonance frequency is an important consideration, as SLBs require an aqueous environment to remain hydrated. In addition to the frequency shift, QCM can also monitor the temporal energy dissipation factor (Δ*D*) on the quartz crystal, making QCM-D (QCM with dissipation monitoring) especially suitable for studying biological samples that can dissipate energy due to their viscoelastic properties. These viscoelastic properties can change, for example, when the bilayer interacts with an analyte or undergoes a phase change; a more structured bilayer structure would dissipate less energy, while a less structured layer would dissipate more energy. By monitoring changes in dissipation, the modulus of the thin film can be determined and the mechanism of interaction with the membrane inferred. In general, lipid bilayers are considered homogeneous soft films, and hence the Voigt–Voinova model is most appropriate for fitting and analyzing such output data (83). The Sauerbrey and Voigt–Voinova models provide ways to evaluate changes in the mass and viscoelastic properties of SLBs, with the abilities to gather both steady-state and time-dependent data, making QCM an especially suitable technique for biosensor applications and characterization of biomolecular interactions between the SLB and interacting analytes.

Over the past two decades, QCM-D has been used to sense and monitor biological interactions with SLB components deposited on the crystal surface. SLBs can be formed from reconstituted lipid vesicles, thereby functionalizing the surface with a layer that mimics the cell membrane lipid bilayer. Depending on the components incorporated into the SLB, various interactions, such as those between pore-forming toxins and SLBs (84, 85) and the tethering of therapeutic lipid-shelled microbubbles to SLBs, can be investigated (81) (Figure 4c). QCM-D is also used to assess binding interactions between SLB components and their binding partners, such as neutravidin proteins binding to SLBs containing biotinylated lipids (86) and glycolipid–antibody binding events (87). In addition, this system is not limited to the use of reconstituted lipid vesicles. In recent years, the Daniel lab and others have demonstrated the formation of SLBs containing native cellular membrane components with QCM-D, extending its applications to probe native membrane interactions with biomolecules (88–92). Similarly, Mohamed et al. (88) demonstrated the formation of SLBs derived from bacterial outer membrane vesicles (OMVs) to sense antibiotic interactions and membrane disruption.

### 4.2. Supported Lipid Bilayer Sensors: Optical Detection

When transparent surfaces are chosen as supports for lipid bilayers, optical methods, such as fluorescence, can be used to characterize SLBs and their interactions with other biological materials. However, at least one component must be fluorescently labeled to report the change, whether that be a component in the membrane or the analyte of interest. One way to label the membrane is to put a fluorescent reporter molecule in the SLB that changes intensity in response to an interaction. Alternatively, the reporter can be on the analyte that interacts with a membrane component. Ideally, when interactions occur at the membrane–analyte interface, changes in fluorescence can be monitored and correlated with specific events and mechanisms.

If binding kinetics or other dynamic membrane sensing information is desired, total internal reflection microscopy (TIRFM) is an optical imaging technique that exploits an evanescent wave to illuminate a very short (~100-nm) vertical region near the SLB surface (93). Thus, TIRFM is a powerful tool to study the interactions occurring near the bulk–SLB interface, as it can effectively eliminate fluorescence from the bulk solution where unbound analytes are located, but outside the evanescent wave excitation field. When paired with single-particle-tracking techniques, TIRFM can probe single-protein dynamics. Richards et al. (94) confirmed the mobility of a single fluorescent protein in a natively derived SLB membrane by using TIRFM. Floyd et al. (95) and, more recently, Chien et al. (80) captured the fusion of influenza virus particles with an SLB model membrane (Figure 4c). In addition to TIRFM, other types of optical techniques such as surface plasmon resonance (SPR) can be used to monitor biological interactions with planar lipid bilayers (96). However, the surfaces used in SPR systems are generally metal, which is less compatible with the formation of SLBs unless the metal surface is first chemically modified. For instance, Hinman et al. (97) coated a gold surface with a thin layer of silica, thereby preserving lipid mobility, and coupled it with an SPR setup to detect various protein–ganglioside interactions.

### 4.3. Supported Lipid Bilayer Sensors: Electrical Detection

When the solid support for SLBs is electrically conductive, the electrical properties of the SLB can be probed. One nondestructive, label-free, and highly sensitive method is electrochemical impedance spectroscopy (EIS). EIS has emerged as a powerful approach to assess biorecognition events occurring near a biomimetic membrane interface residing on a conducting electrode surface (97). This sensing approach consists of applying an alternating voltage through a wide range of frequencies and recording the corresponding current response (88). From these data, the impedance of the electrode–membrane–electrolyte system can be measured and fit to an electronic circuit model to extract the electrical properties (e.g., resistance and capacitance) of the bilayer and provide information about a biorecognition event (98, 99). A commonly used conductive surface is gold, but its drawbacks include a high impedance, which limits sensitivity, and due to gold’s typical surface hydrophobicity, surface modifications are usually required to form an SLB (100, 101).

**Figure 5:**
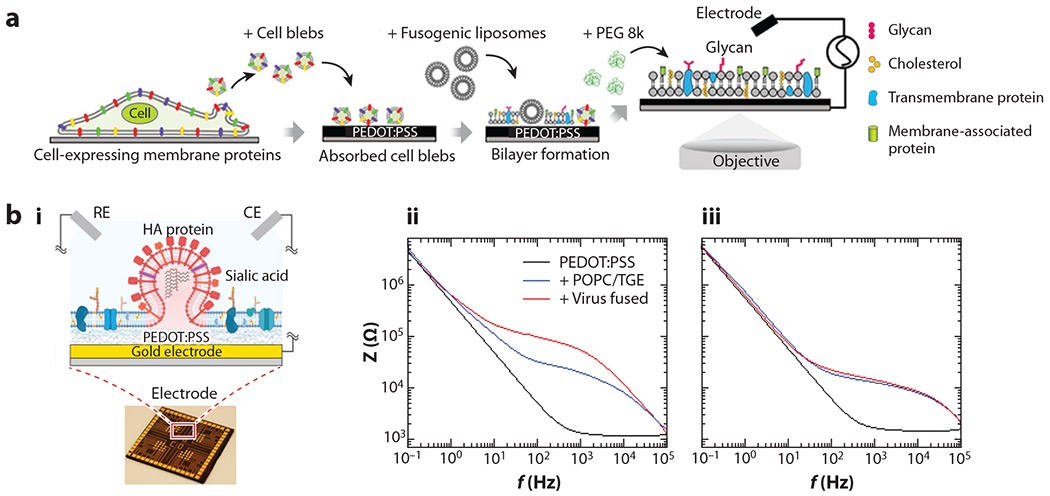
(*a*) Formation of cell-derived blebs, followed by their adsorption onto a PEDOT:PSS surface. Fusogenic vesicles were subsequently added to promote SLB formation incorporating components derived from a cell membrane. Panel *a* adapted with permission from Reference 107; copyright 2020 American Chemical Society. (*b*, *i*) PEDOT:PSS electrode–based electrical sensing platform supporting cell membrane–derived SLB formation. (*ii*, *iii*) Typical electrochemical impedance spectroscopy readouts caused by virus fusion with the SLB. Panel *b* adapted with permission from Reference 109; copyright 2021 American Chemical Society. Abbreviations: CE, counter electrode; PEDOT:PSS, poly(3,4-ethylenedioxythiophene):poly(styrene sulfonate); PEG, polyethylene glycol; POPC, 1-palmitoyl-2-oleoyl-glycero-3-phosphocholine; RE, reference electrode; SLB, supported lipid bilayer.

When EIS is used, it is critical to minimize the detection limit and to isolate membrane impedance changes caused by biological events, like the formation and destruction of SLBs and the sensing of ligand–receptor binding events. A commercially available conductive polymer poly(3,4-ethylenedioxythiophene):poly(styrene sulfonate) (PEDOT:PSS) offers a way to accomplish these goals by significantly lowering system impedance (102), thus maximizing any signal contributions from interactions at or near the SLB interface (103). In addition, the swelling properties of PEDOT:PSS in aqueous solution create a conductive cushion layer that helps preserve the mobility of the constituents, including proteins (94). These features make PEDOT:PSS an ideal material for electrically sensing subtle membrane-associated processes at the SLB surface. However, it is often challenging to rupture zwitterionic phospholipids by vesicle fusion methods on PEDOT:PSS, due to its negatively charged and rougher surface compared to a smooth glass–silica surface (104). To circumvent these issues and enable SLB formation on PEDOT:PSS from a broader array of phospholipids, researchers have explored various approaches, including using a solvent-assisted lipid bilayer (SALB) method (105, 106), doping positively charged lipids into fusogenic vesicles (107, 108), and modifying the PEDOT:PSS surface by using silica nanoparticles (89). By utilizing the SALB methods, our group has fabricated model mammalian and bacterial membranes on PEDOT:PSS to monitor interactions of bacterial toxins and antibiotics with mammalian and bacterial membranes, respectively (106). We have also extended this approach to prepare model synthetic membranes on PEDOT:PSS to study receptor-specific binding events (105). By mixing a small portion of positively charged DOTAP lipids, we have demonstrated the self-assembly of mammalian membranes (BHK) on bioelectronic devices by optimization of the mammalian bleb rupture process on the PEDOT:PSS surface (107) (Figure 5a). The transmembrane protein (P2X2-neon) molecules in the resulting bleb bilayer retain their mobility and orientation while remaining responsive to the presence of ATP. This approach has also been applied to the assembly of SLBs derived from human cells in which blebs, derived from HEK cells expressing TREK-1 transmembrane proteins, were isolated and assembled on a PEDOT:PSS surface (108). In this study, this platform served as a biosensor for drug interactions with the TREK proteins. More recently, native cell membrane–derived SLBs formed on PEDOT:PSS electrodes were utilized to recapitulate the fusion of influenza viruses (Figure 5b) and severe acute respiratory syndrome coronavirus 2 (SARS-CoV-2) pseudoparticles with host cell membrane mimics (109, 110).

In addition to being applied in EIS measurements, PEDOT:PSS is also used in organic electrochemical transistors (OECTs) (102, 103, 111). Its tunable electrical conductance makes SLBs on PEDOT:PSS a perfect platform to study the activity of transmembrane proteins (108, 112). For example, by assembling native membrane SLBs containing TREK-1 proteins on PEDOT:PSS, we have shown TREK-1 channel activity in the presence of an activator and suppressor by monitoring response time changes using OECTs (108).

## 5. APPLICATIONS OF BIOMEMBRANE BIOSENSORS

In recent years, biomembrane biosensors have emerged as useful tools across a multitude of different fields due to the development of new techniques for synthesizing sensors, technologies for measuring readouts, and available toolkits that allow for customization. Here, we highlight different applications of lipid vesicles and SLBs as biosensors and discuss novel techniques, such as CFPS, that provide further opportunities for these platforms.

### 5.1. Health and Disease

Biosensors have garnered utility in clinical settings, drug development, and health care in general and are implemented in disease identification, monitoring, and overall management of human health (113). They also contribute to advances in the field of biomarker detection, especially those associated with cancer (114, 115), diabetes (116), and cardiovascular disease, to name a few examples (117). Samples are typically body fluids like blood, plasma, serum, and saliva, which are cornucopias of diverse compounds with high variability. Capturing and quantifying trace amounts of a target molecule within these intricate sample matrices in the absence of any amplification steps present a formidable task (118). Fortunately, there have been many recent advances in biosensor development with the potential to improve in vitro diagnostics.

#### 5.1.1. Virus detection and screening.

Lipid vesicles have been advantageous for investigating virus–host interactions of enveloped viruses such as influenza A, coronavirus, HIV, and others by integrating viral receptors on the outside of the vesicle and sensing elements on the inside to monitor fusion between vesicles and virus membranes (119). These platforms have identified that cholesterol, for example, is a critical factor for the fusion processes of dengue virus (120), HIV virus, Ebola virus, coronaviruses, and many other known envelope viruses (121). Inspired by the role of cholesterol in viral fusion, Park et al. (122) developed a tunable membrane biosensor for the detection of fusion-competent influenza A virus. During a typical infection, the influenza fusion protein hemagglutinin (HA), located within the viral envelope, binds to sialic acid receptors located in the plasma membrane of the host cell to induce a conformational change in the HA fusion peptide. In turn, the viral and host membranes merge for subsequent replication and infection (123). To simulate this interaction, liposomes decorated with sialic acid receptors and encapsulated FRET dyes were used to fluorescently monitor host cell fusion kinetics of activated influenza viruses. By varying the molar ratio of cholesterol in the membrane, active and inactive influenza A viruses were able to be differentiated, and multiple subtypes of influenza virus could be detected with high specificity. In addition, fusion kinetics were maximized for enhanced sensor performance by tuning the membrane composition, affecting properties like rigidity and fluidity (122). By changing the sialic acid type or membrane composition, this versatile platform has the potential to inform both host tropism changes via receptor screening and the impact of fusion protein mutations on fusion function.

The COVID-19 pandemic prompted the rapid development of biosensing platforms for fast and accurate testing of SARS-CoV-2 and its variants. In response, novel biosensing technologies based on synthetic biomembranes have emerged to contribute to this effort. Ning et al. (124) created a liposome-mediated biosensing platform to fluorescently detect SARS-CoV-2 RNA as an early sign of viral infection with higher accuracy than traditional RT-qPCR techniques. Extracellular vesicles from blood plasma were fused with liposomes encapsulating CRISPR-Cas12a reagents to amplify and detect the SARS-CoV-2 gene, along with a fluorescent probe for readout (124). One of the advantages of this approach is that samples are noninfectious. This is important because infectious samples are more prone to inactivation by harsh environmental factors, such as those found in complex sample matrices like body fluids. As a result, noninfectious samples are more stable and can be detected for a longer period. In addition, no extraction procedures are necessary for obtaining clinical samples. For more information on the use of liposomes specifically for the prevention, detection, and treatment of SARS-CoV-2, we direct interested readers to a full review by Faizal & Amin (125).

Immunosensors use antibodies to detect specific molecular targets as they bind to engineered locations, increasing their popularity in designing sensors for monoclonal antibody (mAb) immunotherapy development. Current methods for evaluating mAbs like enzyme-linked immunosorbent assays (ELISAs) are time-consuming and expensive, but synthetic biosensors offer promising alternatives for antibody screening. Liposomes characteristically have a large surface area, meaning that many receptors can be in the membrane. However, only a small number of the membrane-embedded receptors can be in close contact with the sensing surface because of curvature effects, which can greatly reduce the sensitivity of a device (126). To circumvent this issue, devices with SLBs have been used for increased sensitivity and amenability with techniques using planar surfaces for direct signal readouts. In terms of sensor design, mAbs can be engineered to bind viral spike proteins, inhibiting the interactions between a virus and its respective host cell receptors to prevent viral entry, replication, and infection (127). Batool et al. (128) presented an innovative approach to evaluate the effectiveness of mAbs as potential antiviral therapies against SARS-CoV-2 by functionalizing a plasmonic sensor with an SLB containing spike protein receptors (ACE2). In another study by Zhou et al. (129), an SLB containing the ACE2 receptor was functionalized onto an extended gate electrode with the capability of monitoring the binding of SARS-CoV-2 spike proteins to ACE2 receptors in real time. This lab-on-chip-style biosensor was able to demonstrate specificity in screening for COVID-19 blocking drugs, which presents opportunities for the SLB sensors to be implemented as screening platforms for a wide range of antiviral drug therapeutics (129).

#### 5.1.2. Antibiotic–pathogen interactions.

Bacteria are becoming increasingly resistant to existing antibiotics such that antibiotic resistance is outpacing drug development. As a result, the cost of drug development continues to rise; infections are more difficult to detect and expensive to treat; and in some cases, certain infections have no effective antibiotic treatments, causing and risking widespread outbreaks (130). Biomembrane sensors offer two useful tools for these challenges: (*a*) for the development of novel antibacterial agents that target the membrane to permeabilize or disrupt it in some manner and (*b*) for the development of pathogen detection wherein the membrane mimics the host and interactions with the pathogen can be characterized for identification.

The current gold standards for pathogen detection include PCR and enzyme immunoassays like ELISA, but these techniques are costly and low throughput (131). Hence, synthetic biosensors have been developed to identify pathogenic organisms precisely and rapidly through carefully engineered molecular recognition and signaling mechanisms. For example, colorimetric PDA-incorporating vesicles, described in sections 3.2 and 3.3, were used to identify multiple strains of bacteria at the genus and species levels via a color change. It was discovered that lipid membrane composition significantly influenced the sensitivity of detection for different bacteria strains, exhibiting a unique chromatic fingerprint for each individual strain (132).

SLBs have demonstrated potential in electrochemical sensing for the label-free detection of target molecules like cholera toxin and antibiotic compounds (106, 133). The antibiotic resistance of gram-negative bacteria presents many challenges, making them sought-after targets for biosensors. As a result, synthetic electrical sensing platforms to study the interactions between antibiotics and bacterial outer membranes have been developed. An example that nicely highlights this application is a platform made from SLBs derived from the OMVs of *Escherichia coli* formed on a PEDOT:PSS electrode to enable electrochemical sensing of membrane disruption. The platform reported bacterial membrane–antibiotic interactions with multiple antibiotics: polymyxin B, bacitracin, meropenem, and vancomycin. In addition, this platform is compatible with OMV bilayers derived from several bacteria: *Pseudomonas aeruginosa*, *Acinetobacter baumannii*, and *Enterobacter cloacae*, which are among a group of pathogens that are the leading causes of global infections (88, 134, 135). These novel biosensor designs offer ways to screen membrane-disrupting compounds that could open new treatment options in the future.

### 5.2. Environmental Monitoring

As the human population and industrialization continue to rise, our air, water, and soil are becoming increasingly contaminated with both organic and inorganic pollutants, causing danger to the health of our biosphere and its inhabitants. As of now, most water and soil analyses are based on manual sampling with costly and laborious sample analysis. For a robust biosensor capable of detecting contaminants with high specificity in harsh real-life environments, several features are highly desired: (*a*) fast response times, (*b*) high sensitivity, and (*c*) prolonged stability for long monitoring times. In addition, biosensors should exhibit reproducibility, transportability, and reusability when possible (136). Designing a durable biosensor that meets all those requirements is not trivial. Although some early whole-cell sensing technologies have been used for environmental monitoring (137, 138), whole cells can still present other challenges associated with lack of stability and potential deactivation in the presence of pollutants often contained in the testing samples (138). Fortunately, researchers have made great strides toward the development of lipid-based biosensors for use in environmental monitoring, as they have the potential to meet all the criteria listed above, are nonliving, and cannot propagate.

**Figure 6:**
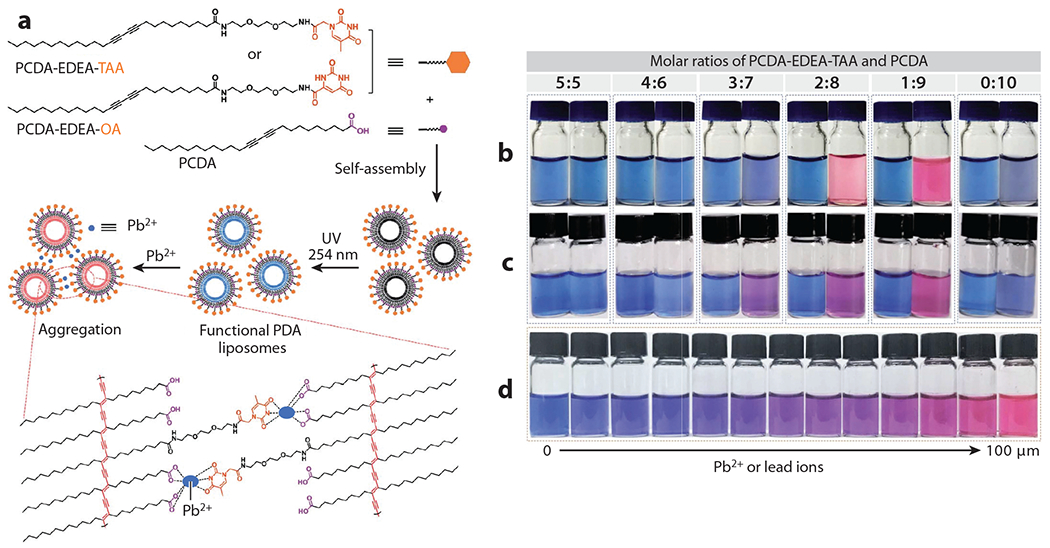
(*a*) The functionalized PDA monomers self-assemble and, once polymerized by UV light, result in a PDA-based liposome with a blue color. The PDA liposomes then bind to lead ions, inducing a conformational change in the PDA polymer backbone and aggregation of the polymer chains. This aggregation shifts the absorption spectra, resulting in a color change from blue to red. (*b*,*c*) Color change to PDA liposomes before and after the addition of 100 μM Pb^2+^, where the PDA liposomes were made with varying ratios of (*b*) PCDA-EDEA-TAA and PCDA and (*c*) PCDA-EDEA-OA and PCDA. (*d*) Change in color of liposomes made of PCDA-EDEA-TAA and PCDA at a 1:9 ratio in the presence of increasing Pb^2+^ concentrations. Figure adapted with permission from Reference 144 (CC BY-NC 3.0). Abbreviations: EDEA, ethylenedioxy-bis-ethylamine; OA, orotic acid; PCDA, 10,12-pentacosadiynoic acid; PDA, polydiacetylene; TAA, thymine-1-acetic acid; UV, ultraviolet.

Pollutants, such as heavy metals from industrial waste, can be detrimental to many organ systems if ingested by humans in even the smallest amounts (139). For example, lead (Pb^2+^) is one of the most infamous heavy metal contaminants in terms of human health, causing millions of deaths globally per year (140). Lead inhibits various key enzymes used for the synthesis of hemoglobin and reduces the life span of erythrocytes by increasing the fragility of cell membranes, making lipid sensors opportune platforms for lead detection and study. Over the years, many reports of PDA liposome–inspired sensors have emerged for the detection of heavy metals like lead (141) and mercury (142). Recently, a dual colorimetric and fluorometric sensor was developed for the detection of Pb^2+^ in a rapid (10-s) and sensitive [limit of detection (LOD)=0.1 μM] manner. This sensor design took advantage of the fluorescently dependent binding interactions between Pb^2+^ ions and a phenylboronic acid moiety that was functionalized onto PDA liposomes (143). Chen et al. (144) developed a similar highly selective Pb^2+^ sensing platform with functionalized PDA liposomes, resulting in Pb^2+^ detection limits as low as 25 nM; in addition, a visible color change can be observed by the naked eye at 20 μM in real-world water samples. Thymine-1-acetic acid or orotic acid groups can be integrated into PDA liposomes with 10,12-pentacosadiynoic acid to create binding sites for Pb^2+^ ions. Upon exposure to UV light, polymerization is induced, and the liposome solution turns a deep blue color and drastically changes in fluorescence (144) (Figure 6). In another example, De Leo et al. (145) presented a novel method for removing methylene blue, a common water pollutant from dyes in the textile industry, using liposome–polydopamine microspheres, which are liposomes encapsulated inside of a polydopamine shell. The liposomes bind to available methylene blue molecules to induce a detectable chromatic signal, which results in high adsorption capacity and rapid removal of methylene blue from water samples. Additionally, these liposomal microspheres can be obtained in the absence of organic solvents, offering a versatile and totally green approach for water purification applications (145).

### 5.3. Food Safety and Quality Control

Conventional techniques for food analysis include various forms of chromatography or mass spectrometry, which are generally carried out at the end of the production process. Although highly sensitive, these methods are costly and time-consuming, require large sample volumes, and demand highly skilled operators. Biosensors employing liposomes have proven to be potentially effective solutions to the current challenges in rapidly and effectively assessing the quality and safety of food. On many accounts, the versatility of liposome-based biosensors has been demonstrated with the detection of various metals, toxins, and pathogens in both food and water samples, as well as through their role in food spoilage indication (146, 147).

#### 5.3.1. Pathogen detection in food.

In addition to being used in environmental applications, PDA liposome–based biosensors are relevant in the food sector to monitor food contaminants for consumer safety. There have been many reports of using PDA liposomes as sensors for the detection of dangerous pathogens such as *Staphylococcus aureus*, *Salmonella choleraesuis*, *E. coli*, and more in food samples (148). Nie et al. (149) presented a novel approach for the detection of patulin, a harmful mycotoxin commonly found in fruits and fruit-based products, employing a PDA liposome–based sensor. Inspired by the known reaction between thiol groups and patulin, the approach involved liposomes encapsulating coumarin-6, a fluorescent signal probe. These liposomes were decorated with terminal thiol groups and were combined with patulin-containing samples for sensing analysis; additionally, magnetic nanoparticles with an affinity for liposomes with unreacted thiol groups were introduced into the samples for simple purification by magnetic field. High selectivity and sensitivity toward patulin (LOD = 0.033 ng mL^−1^) were demonstrated for real-world apple and grape juice samples. The accuracy was verified by liquid chromatography–tandem mass spectrometry (149).

#### 5.3.2. Toxicant detection in food.

Food toxicants are also a concern in protein-rich food products. For example, histamine is a biogenic amine involved in the immune response produced by bacteria in spoiled fish, and if such fish are consumed in high enough concentrations, a foodborne illness termed scombroid poisoning results. Biosensors for histamine detection have existed for decades, but most are limited by the need for extensive pretreatment of samples or large volumes and for costly equipment (150). Nonetheless, the use of artificial cells like liposomes is emerging for histamine detection (151). Bajpai et al. (152) developed an immunosensor based on antigen–antibody interactions for fluorescent detection of histamine molecules. Liposomes encapsulating self-quenching sulforhodamine B dye were conjugated with antihistamine antibodies that specifically bind to present histamine molecules. Histamine from various real-world samples (fresh mackerel fish, canned tuna and salmon, ground red meat, and prepackaged salad) was detected in trace (2–3-ppb) amounts. Additionally, traditional detection techniques, like liquid chromatography–mass spectrometry, were outperformed by this sensing technology, where traditional methods were limited to detecting >1 ppm of histamine in various food samples (152).

Recent advances in synthetic nanotechnology have led to the development of biomembrane biosensors based on immobilization of enzymes, antibodies, and receptors within lipid films, improving biosensor sensitivity for application in the food industry. Metal-supported lipid membranes have been used, but few efforts have implemented SLBs (153). The constant hydration requirement of SLBs can pose challenges in the design of biosensors for food quality monitoring, as maintaining optimal hydration levels is not always practical for various food sample matrices. As a result, researchers have begun to focus on developing air-stable lipid membranes for food quality monitoring and many other relevant applications (154). As research continues to address the challenges of SLBs, SLBs have the potential to become a more widely used technology for food safety and quality monitoring applications.

### 5.4. Emergence of Cell-Free Biosensors

The combination of CFPS technology and synthetic biology has the potential to create a new generation of biosensing tools that can be used to improve environmental monitoring, health care, and many other fields. Synthetic biology techniques that have been shown to improve cell-based sensing performance can also be applied to cell-free biosensors (155), expanding the range of applications.

Biosensors assembled using CFPS techniques are advantageous for studying virus–cell membrane interactions, because they are not affected by artifacts like mutations that come with passaging cells repeatedly, and there is no need for specialized biocontainment, due to lack of genetically engineered microorganisms (156). Such liposomal biosensors have successfully incorporated CRISPR-Cas9 technology and have already demonstrated highly sensitive detection of pathogenic sequences in dengue and Zika virus RNA genomes (157).

CFPS sensing platforms hold exciting prospects as tools for detecting clinically relevant biomarkers in infectious disease. For example, *P. aeruginosa* is a gram-negative bacterium that can cause a variety of respiratory infections. *P. aeruginosa* behavior is dependent on a process termed quorum sensing, which involves the production and detection of small signaling molecules that trigger the production of virulence factors from bacteria. In a study by Wen et al. (158), a biosensor was presented for detecting autoinducers in *P. aeruginosa*–infected samples. The biosensor consisted of a cell-free DNA-encoded circuit that produces a GFP signal in the presence of the *P. aeruginosa*–specific autoinducer 3OC12-HSL. The circuit was encapsulated in a liposome, protecting it from degrading in harsh environments, like those in clinical samples. Nanomolar detection of 3OC12-HSL was achieved in sputum samples from patients infected with *P. aeruginosa*. This work demonstrates the potential of cell-free biosensors for monitoring progression of lung infections and many other types of infections by tuning the system toward various biomarker detection (158). CFPS proteoliposomes have also been used to study the OprF membrane protein interactions of *P. aeruginosa* (159).

**Figure 7:**
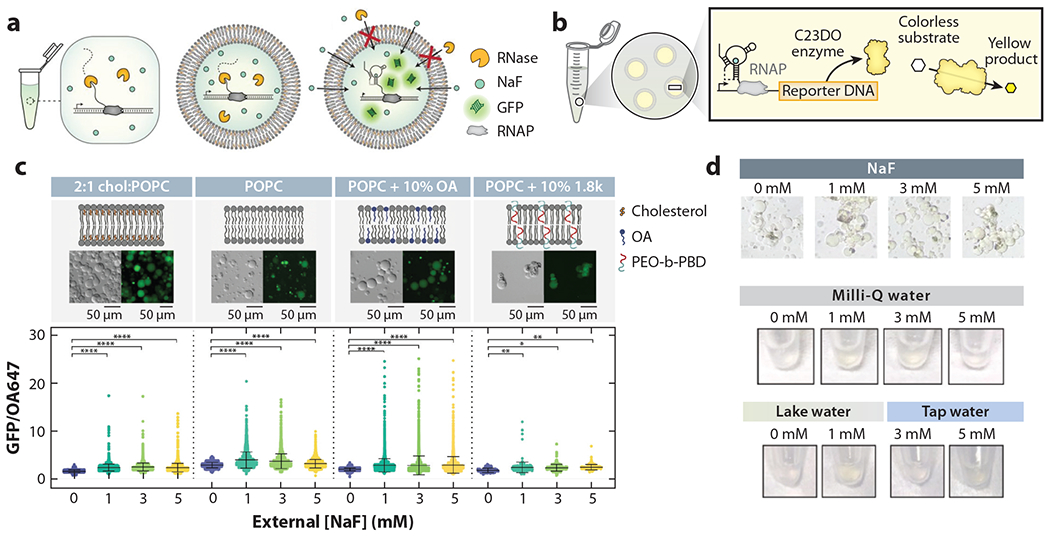
(*a*) The riboswitch technology is degraded by RNase A under both bulk conditions and when the RNase A is intentionally co-encapsulated with the cell-free reaction components. However, reactants are protected from RNase A when encapsulated in a vesicle. (*b*) After encapsulation, the activation of the riboswitch results in the expression of the C23DO enzyme, creating a yellow product. (*c*) By manipulating membrane composition, the riboswitch can be tuned for optimal fluoride detection based on GFP/OA647 fluorescence after the addition of increasing concentrations of fluoride. ****, *P* ≤ 0.0001; **, *P* ≤ 0.01; *, *P* ≤ 0.05; ns, *P* > 0.05. (*d*) Colorimetric changes of Milli-Q water, local lake water, and tap water samples containing the riboswitch technology after the addition of NaF can be visualized by a microscope eye piece and by the naked eye. Figure adapted with permission from Reference 74 (CC BY-NC 4.0). Abbreviations: GFP, green fluorescent protein; OA, orotic acid; PEO-b-PBD, poly(ethylene oxide)-*b*-poly(butadiene); POPC, 1-palmitoyl-2-oleoyl-glycero-3-phosphocholine; RNAP, RNA polymerase.

The combination of SLBs and CFPS leads to a highly customizable biosensor amenable to various surface characterization techniques. In one instance, CFPS has been implemented in SLB-based biosensors to investigate ion channel behavior with electrochemical sensing platforms, as our lab has shown with MscL (mechanosensitive channel of large conductance) (160). The bottom-up approach of CFPS–SLB platforms allows researchers to mimic cellular interactions for artificial cell construction and potentially even organs by starting with the simplest components and adding complexity in a stepwise manner. For example, the placenta is a critical component for transport of nutrients to a fetus; thus, any exposure to harmful agents and toxicants can severely affect fetal development. To address this, CFPS–SLB technologies have been used to create models of placental cells that mimic the lipid composition at varying stages of pregnancy, creating a biosensor useful for screening through molecular interactions that can help identify harmful toxins throughout pregnancy (161). As briefly introduced in Section 3.4, cell-free biosensing technology has now begun to reach the environmental sector, where a customizable riboswitch-based CFPS biosensor was created to detect fluoride in real-world water samples, the first of its kind (74) (Figure 7). Overall, CFPS and SLBs are emerging as a new complement and potentially an alternative to current cell-based biosensing applications.

## 6. CONCLUDING REMARKS AND OUTLOOK

The field of biomimetic membrane sensing is continuously being improved and incorporated into various applications. On one hand, the incorporation of CFPS into membrane sensors allows entirely synthetic assemblies to be more biocompatible, expanding the variety of proteins that can be used as sensing elements or as chaperones to facilitate sensing. On the other hand, the continuous development of new tools and assays for identifying, isolating, and manipulating biologically derived vesicles, as opposed to the synthetic ones described here, can potentially enable us to integrate all the design principles of artificial sensors into those that are entirely biologically relevant. We envision that as we expand the toolbox to more biomacromolecules, biomimetic sensors will transition into the gold standard for sensing, eventually offering selectivity and specificity unrivaled by other sensors.
